# An in silico pipeline approach uncovers a potentially intricate network involving spike SARS-CoV-2 RNA, RNA vaccines, host RNA-binding proteins (RBPs), and host miRNAs at the cellular level

**DOI:** 10.1186/s43141-022-00413-5

**Published:** 2022-09-06

**Authors:** Massimiliano Chetta, Marina Tarsitano, Maria Oro, Maria Rivieccio, Nenad Bukvic

**Affiliations:** 1 AORN A. Cardarelli–Dipartimento delle Tecnologie Avanzate Diagnostico-Terapeutiche e dei Servizi sanitari-U.O.C. Genetica Medica e di Laboratorio, Via A. Cardarelli 9, 80131 Napoli, Italy; 2 AOUC “Policlinico di Bari”-UOC Lab. di Genetica Medica, Piazza Giulio Cesare 11, 70124 Bari, Italy

**Keywords:** SARS-CoV-2, In silico analysis, Exogenous RNA, RNA-binding proteins (RBPs), Human miRNAs, Host cell network modulation

## Abstract

**Background:**

In the last 2 years, we have been fighting against SARS-CoV-2 viral infection, which continues to claim victims all over the world. The entire scientific community has been mobilized in an attempt to stop and eradicate the infection. A well-known feature of RNA viruses is their high mutational rate, particularly in specific gene regions. The SARS-CoV-2 S protein is also affected by these changes, allowing viruses to adapt and spread more easily. The vaccines developed using mRNA coding protein S undoubtedly contributed to the “fight” against the COVID-19 pandemic even though the presence of new variants in the spike protein could result in protein conformational changes, which could affect vaccine immunogenicity and thus vaccine effectiveness.

**Results:**

The study presents the findings of an in silico analysis using various bioinformatics tools finding conserved sequences inside SARS-CoV-2 S protein (encoding mRNA) same as in the vaccine RNA sequences that could be targeted by specific host RNA-binding proteins (RBPs). According to the results an interesting scenario emerges involving host RBPs competition and subtraction. The presence of viral RNA in cytoplasm could be a new tool in the virus’s armory, allowing it to improve its chances of survival by altering cell gene expression and thus interfering with host cell processes. In silico analysis was used also to evaluate the presence of similar human miRNA sequences within RBPs motifs that can modulate human RNA expression. Increased cytoplasmic availability of exogenous RNA fragments derived from RNA physiological degradation could potentially mimic the effect of host human miRNAs within the cell, causing modulation of the host cell network.

**Conclusions:**

Our in silico analysis could aid in shedding light on the potential effects of exogenous RNA (i.e. viruses and vaccines), thereby improving our understanding of the cellular interactions between virus and host biomolecules. Finally, using the computational approach, it is possible to obtain a safety assessment of RNA-based vaccines as well as indications for use in specific clinical conditions.

## Background

SARS-CoV-2 has enlarged the family of coronaviruses of human interest by demonstrating increased virulence and the ability to cause serious disease forms [1]. Following a relaxation of the strict emergency protocol (lockdown) in the summer months of 2020, many countries experienced the reemergence of infections characterized by rapid spread and identification of specific genetic variants [2].

The emergence of new strains has been linked to new pathogenic variants in the S protein, including missense, deletion, and insertion mutations [3].

In January 2020, China reported the first SARS-CoV-2 variant in spike glycoprotein, which was an amino acid change at position 614. (p.D614G). In May 2020, the variant p.D614G was isolated in 20% of the Brazilian population, while it was found in almost all infected people in February 2021 [3].

A new variant (p.N501Y) called B.1.1.7 was discovered in the UK in October 2020, and a new variant called B.1.351 was discovered in South Africa in December of the same year (501Y.V2). The other variant in position 484 (p.E484K) was also described in both new strains. Variants of the p.K417N, p.E484K, and p.N501Y were discovered in Japan, the USA, France, and Italy the following month [4].

In December 2020, a new strain known as (P1) B.1.1.28 was discovered in Brazil, and 1 month later, a new strain known as P2 was discovered in Rio de Janeiro, but it was less aggressive than P1 [5]. South Africa alerted the World Health Organization (WHO) on November 24, 2021, that a new SARS-CoV-2 variant, B.1.1.529, had been discovered. At least 30 amino acid substitutions, 3 small deletions, and 1 small insertion are found in the Omicron variant’s spike protein, with 15 of the 30 amino acid substitutions occurring in the receptor-binding domain (RBD) [6]. The World Health Organization (WHO) has proposed categorizing variants as variants of concern (VOC) and variants of interest (VOI) [7].

Some VOI variants have been linked to receptor-binding conformational changes, reduced neutralization by antibodies produced against previous infection or vaccination, and reduced treatment efficacy. The class of VOC, on the other hand, includes variants that increase transmissibility (e.g., increased hospitalizations or deaths), decrease neutralization by antibodies generated during previous infection or vaccination, and prevent proper diagnostic classification [8].

VOI strains include Eta (B.1.525), Iota (B.1.526), and Kappa (B.1.617.1, B.1.617.3), while VOC strains include Alpha (B.1.1.7), Beta (B.1.351, B.1.351.2, B.1.351.3), Delta (B.1.617.2, AY.1, AY.2, AY.3), and Gamma (P.1, P.1.1, P.1.2). In this group, Omicron strain B.1.1.529 has also been introduced [7]. The focus on new emerging variants and the severe pandemic situation has shifted attention away from immunological data collection. Non-specific assays developed for detecting IgG and IgM antibodies directed against spike (S) and/or nucleocapsid (N) proteins are frequently used to collect antibody response data, resulting in inconsistent results [9].

These tests revealed a rapid decline in antibody titer, particularly in people who had an asymptomatic or paucisymptomatic infection. However, without accounting for the more complex mechanism in which memory cells play a role in their ability to reactivate, this decline has also been observed in those who have been vaccinated (B and T lymphocytes) [10, 11]. The observation of improved B cells and antibody responses in seropositive individuals following infection or vaccination led to the recommendation of additional vaccination dose [12].

COVID-19 has the potential to become an endemic disease capable of spreading throughout the human population in a broad and recurrent manner, similar to how influenza viruses do [13]. This is based on the lifespan of immunity as well as control measures. Although the vaccine has proven to be highly effective and safe, further research into possible interactions between the exogenous RNA introduced into the cells and any cytoplasmic proteins, as well as the potential role of RNA degradation products (i.e., small non-coding RNA) that could mimic microRNA's effect inside receiving cells, may be required.

MiRNAs are non-coding, single-stranded RNA molecules with about 20–22 nucleotides that are found in plants, animals, and even some DNA viruses [14–16]. They play an important role in transcriptional and post-transcriptional regulation of gene expression. By interacting with complementary sequences on target messenger RNA (mRNA) molecules, miRNAs are incorporated into the RNA-induced silencing complex (RISC) and cause gene silencing [16]. This bond prevents the target molecules from being translated or degraded. Although it is unclear whether viral RNA can generate miRNA and thus interfere with host gene expression regulation, it is possible [17].

By modulating parts of the machinery of host cells and inducing epigenetic and epitranscriptomic alterations of genes, evidence of the presence of viral mechanisms capable of directly interacting with genomic RNA has recently been reported in the literature [17]. Positive-strand RNA viruses (+ssRNA viruses) can bind to host RNA-binding proteins (RBPs) and use them to perform a variety of tasks during viral replication (e.g., assisting in the assembly of membrane-bound replicase complexes, regulating replicase activity, and increasing the stability of viral RNAs that escape cellular RNA degradation pathways) [18].

A scenario of host transcriptional factors (TFs) competition and subtraction has also been reported [17]. The simple presence of viral RNA in the cell cytoplasm could represent an additional tool in the virus’s “hands,” allowing it to improve its chances of survival by altering cell gene expression and thus interfering with host cell processes [18, 19].

Based on these hypotheses, we used our pipeline to look for possible conserved RNA-binding domains sequences recognized specifically by host RBPs in all RNA sequences coding for the S protein, and then in the vaccine sequence. In addition, the in silico analysis was extended to focus on particular RNA sequences, capable to bind within the RBPs-binding motifs, than effects should be similar to role of human miRNAs. Namely, an incensement in the cytoplasmic availability of these small RNAs could mimic the effect of miRNAs within the cell by modulating the host cell’s network. This could lead to temporary delay of physiological degradation due to major “resistance” of RNA fragments bind to RBPs.

## Methods

**Fig. 1:**
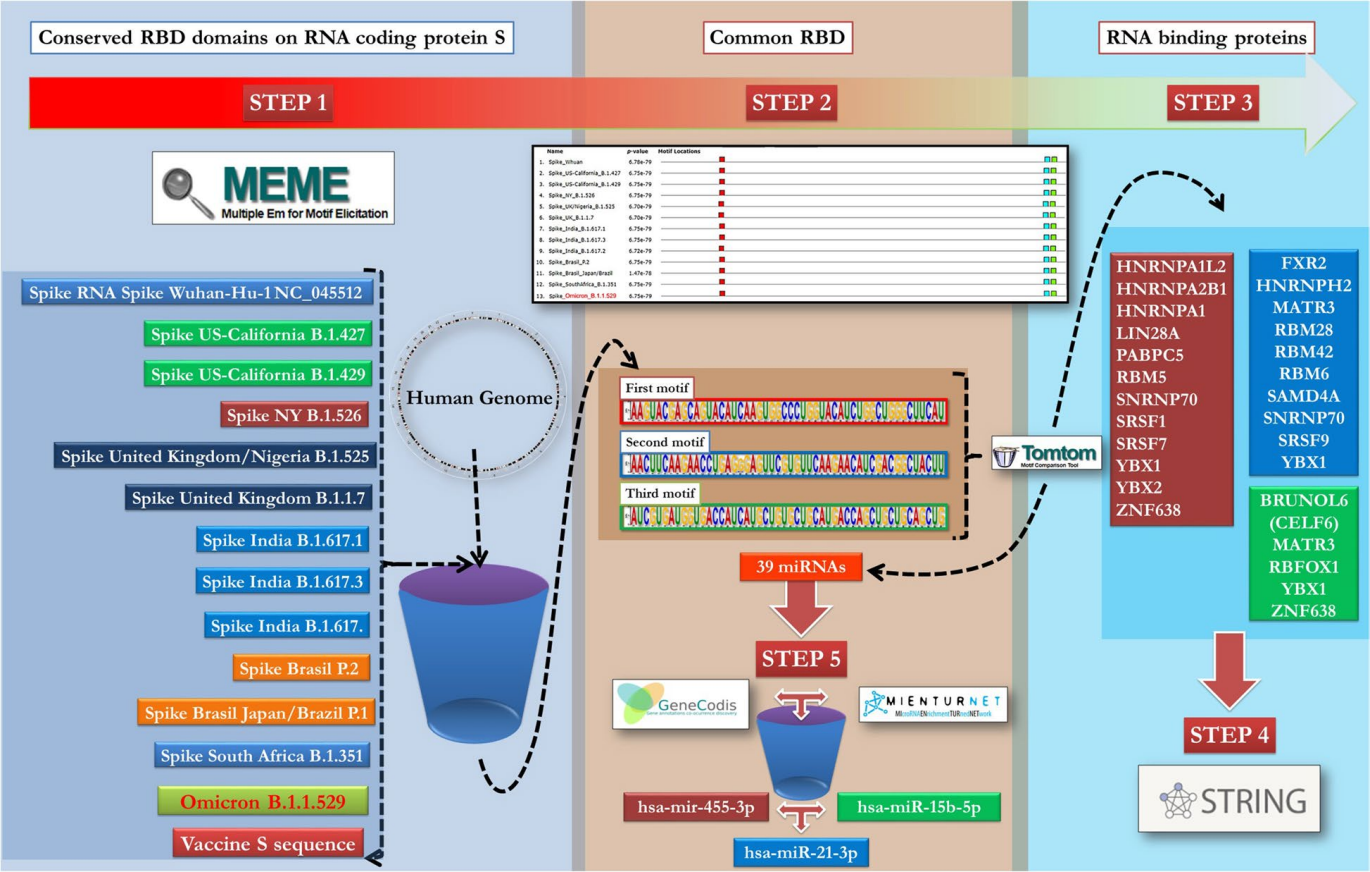
The analysis pipeline's main steps and bioinformatics tools. There are five steps in the pipeline: Step 1: Identify conserved motifs by aligning and analyzing all RNA coding spike protein and vaccine sequences. MEME output is the second step. The analysis reveals the presence of three distinct motifs across the entire S sequence (box red, light blue, and green). The identified nucleotide sequences are listed below the alignment. Step 3: The sequences were fed into TomTom, which compared the newly discovered motifs to the database of *Homo sapiens* RNA-binding motifs. For all motif queries, a list of Human RNA-binding proteins was obtained (box red, light blue, and green). Step 4: STRING is used to look for RBP protein interactions in metabolic pathways. Step 5: The same motifs are used as a query for TomTom to find mature microRNAs within conserved sequences using the micro-RNA database. The list of 39 miRNA was obtained and used in GeneCodis4 and MIENTURNET as a query. The results of the two software programs are combined to infer possible shreds of evidence of significant concurrent annotations (computational or experimental), with those that are significantly enriched being evaluated

The analysis pipeline consisted of five main steps that are carried out using various online bioinformatics tools:

1. Examination of all SARS-CoV-2 Spike RNA-coding spike proteins derived from the most well-studied strains (Spike Wuhan-Hu-1 NC_045512, SpikeUS-California B.1.427, Spike US-California B.1.429, Spike NY B.1.526, Spike United Kingdom/Nigeria B.1.525, Spike United Kingdom B.1.1.7, Spike India B.1.617.1, Spike India B.1.617.3, Spike India B.1.617.2, Spike Brasil P.2, Spike Brasil Japan/Brazil P.1, Spike South Africa B.1.351, Spike B.1.1.529), as well as the full-length spike protein-encoding mRNA of Pfizer-BioNtech, Moderna, and CureVac vaccines containing proline substituted in positions 986 and 987 (p.K986P and p.V987P), were used to find conserved motifs on RNA sequences. MEME (multiple EM for motif elicitation) was used to analyze *.FASTA RNA sequences with default parameters. As shown in step 2 of Fig. 1, the analysis generates a list of highly conserved sequences sorted by *p* value [20].
2. All of the obtained motifs were used as queries in Tomtom ((https://meme-suite.org/meme/tools/tomtom)), a MEME suite tool that compared the newly discovered motifs to RNA-binding motifs in *Homo sapiens*. Tomtom ranked the motifs in the database and used RNA-binding proteins to recognize the RNA motifs, which included 205 genes from 24 different eukaryotes. Because the sequence specificities of RNA-binding proteins are highly conserved throughout evolution, the recognition preferences of a large fraction of RNA-binding proteins can be inferred from the sequence of their RNA-binding domain. For all motif queries, a list of human RNA-binding proteins with the common conserved domain was obtained. Table 1 lists all of the human sequences that RBP target [21].
3. Tomtom selected the miRBase v22 *Homo sapiens* (hsa) database using the same conserved motifs as a query. The rna2meme utility was used to find mature microRNAs with RBPs-like motifs. Matches in the seeding region (positions 2 through 7) are heavily favored. Table 1 lists all of the human microRNAs [21].
4. STRING (Search Tool for the Retrieval of Interacting Genes/Proteins-https://string-db.org/ /), a bioinformatics tool and biological database of known and predicted protein–protein interactions. The STRING database incorporates data from a variety of sources, including experimental data, computer prediction approaches, and publicly available text collections. Using a variety of functional classification methods such as GO, Pfam, and KEGG, the resource additionally highlights functional enrichments in user-provided protein lists. (Fig. 3) [22].
5. The list of possible miRNA obtained through Tomtom analysis is used as a query for two different programs: MIENTURNET (http://userver.bio.uniroma1.it/apps/mienturnet/), an easy-to-use web tool that predicts miRNA-target interactions using data from TargetScan and miRTarBase, and GeneCodis4 ((https://genecodis.genyo.es/) ), which integrates multiple sources to provide functional enrichment analysis. From the input list of mature miRNAs, we combine the results of two different software to infer possible evidence of significant concurrent annotations (computational or experimental) and evaluate those that are significantly enriched [23, 24].

**Table 1:**
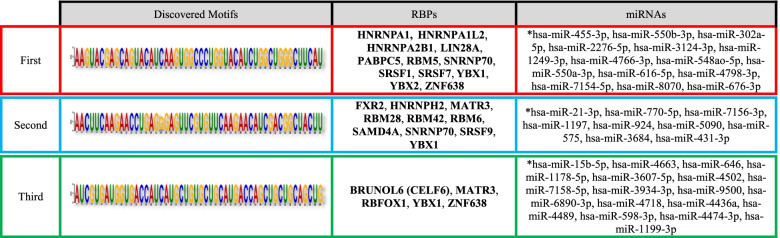
The in silico sequences of the three motifs identified in S protein-encoding mRNA, the RBPs capable of binding the sequences, and the miRNAs with similar sequences to the motif are reported in the table

## Results

### The results of an integrated system biology analysis on RBPs

**Fig. 2:**
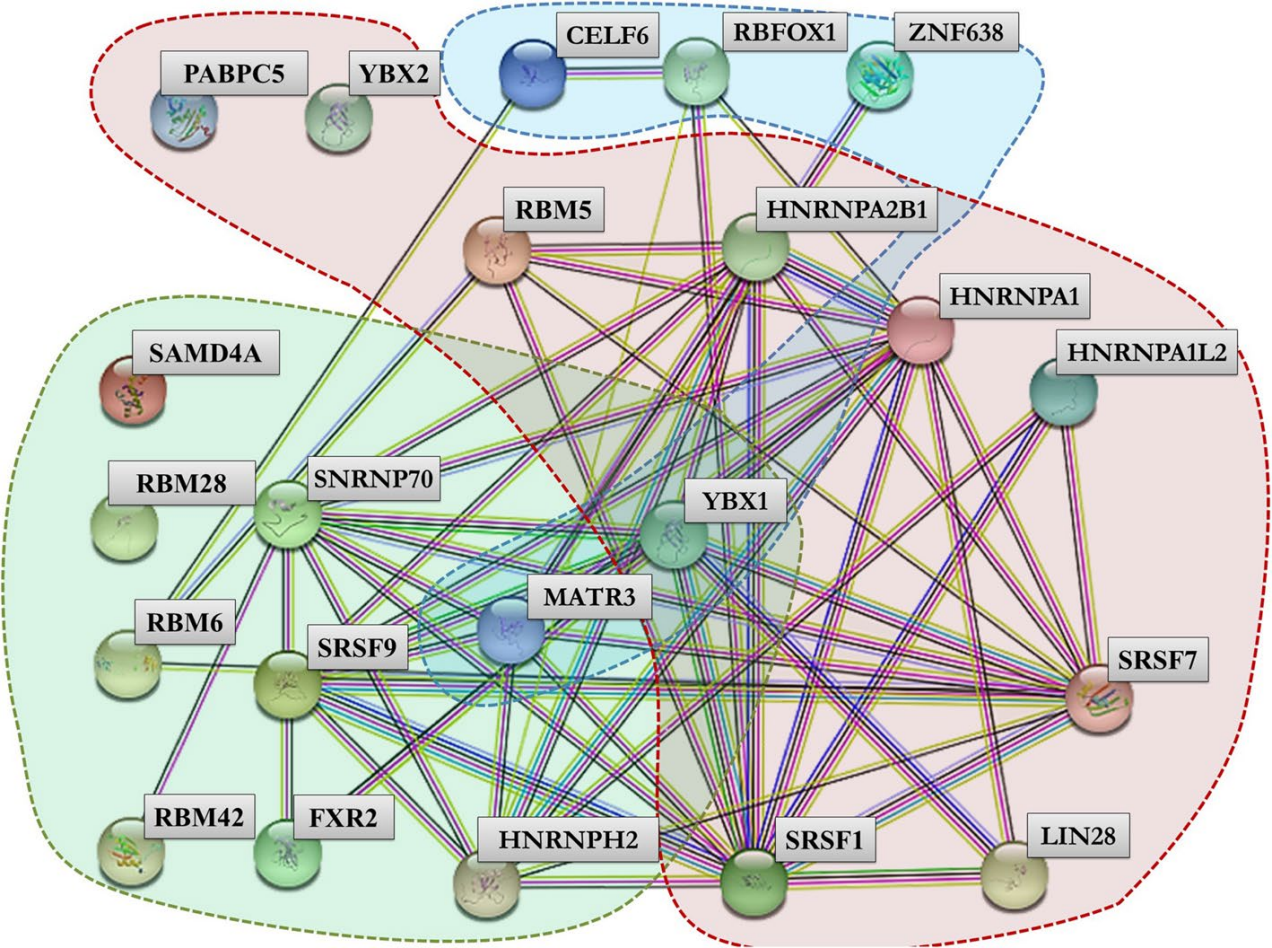
STRING output showing the possible connection between RBPs. The specific RBPs capable of binding the identified target are highlighted in red, light blue, and green

The use of our pipeline in the analysis of all SARS-CoV-2 S protein-encoding mRNA from reported variants in literature as well as the mRNA vaccines recognize by the WHO, revealed the presence of RBP-targeted motifs that are conserved. Our method identified 22 possible RBPs motifs, 18 of which interact closely, as shown in Fig. 2.

Some RBPs can bind the spike RNA multiple times. The protein Y-box-binding protein 1 (YBX1), in particular, binds to all three RBP motifs identified (sequences motifs are shown in Fig. 1). YBX1 is involved in a variety of cellular functions, including transcription and translation control, pre-mRNA splicing, DNA repair, and mRNA packaging. This protein is also found in mRNP complexes and may play a role in microRNA processing. The possibility of YBX1-binding to multiple motifs distributed across the mRNA coding Spike could reduce cytoplasmic protein availability [25, 26].

Knocking down YBX1 expression inhibits K562 leukemic cells proliferation and myeloid differentiation, according to scientific literature. Through the GSK3B/Cyclin D1/Cyclin E1 pathway, YBX1 depletion induces apoptosis and decreases cyclin D1 and cyclin E1 protein expression, resulting in a reduction in Pancreatic Ductal Adenocarcinoma growth [27].

MATR3 (Matrin 3), ZNF638 (Zinc finger protein 638) and SNRNP70 (Small Nuclear Ribonucleoprotein U1 Subunit 70) proteins may also bind multiple spike mRNA motifs. The second and third RBP motifs are binded by MATR3, the first and third RBP motifs are binded by ZNF638, and the first and second RBP motifs are binded by SNRNP70 (Fig. 2). Matrin 3 is an RNA/DNA-binding protein with multiple functions in gene expression regulation, including stabilizing target RNAs, supporting transcription factor activity, and modulating chromatin architecture [28].

Matrin 3 is also involved in the regulation of the transcriptional and translational networks, which control cell growth and proliferation, most likely through the formation of nuclear protein complexes that modulate pro and antiapoptotic signaling pathways. Reduced cell proliferation, which causes endothelial necrosis, is caused by matrin 3 downregulation [29].

ZNF638 inhibition reduces adipocyte differentiation and expression of adipocyte-specific genes. ZNF638 promotes adipogenesis by acting as a transcriptional co-factor of CCAAT/enhancer-binding protein (C/EBP), which leads to the expression of the peroxisome proliferator-activated receptor (PPARG), which controls adipocyte differentiation [30].

SNRNP70 is a nuclear ribonucleoprotein that binds to spliceosomal RNA U1 and is a key component of the spliceosome (U1snRNP). SNRNP70’s cytoplasmic pool plays an important role in nerve-dependent acetylcholine receptor (AChR) clustering and neuromuscular synaptogenesis. SNRNP70 can modulate the transcriptome composition independently of its nuclear activities, according to current evidence [31].

The interaction of HNRNPA1 (heterogeneous nuclear ribonucleoprotein A1), HNRNPA1L2 (heterogeneous nuclear ribonucleoprotein A1 like 2), and HNRNPA2B1 (heterogeneous nuclear ribonucleoprotein A2/B1) is a key component of the network. The first motif can be binded by HNRNPA1, while the second motif can be binded by HNRNPH2 (Heterogeneous Nuclear Ribonucleoprotein H2). These proteins are part of a family of heterogeneous nuclear ribonucleoproteins (hnRNPs) that are involved in nucleoplasmic pre-mRNA processing, as well as mRNA maturation and transport [32].

These proteins’ ability to recognize and bind other RNA molecules, such as hairpin-containing primary transcripts (pri-miRNA), appears to be very intriguing. By increasing the affinity of DGCR8 for primary miRNA transcripts, this specific bind process promotes pri-miRNA processing [33].

HNRNPA1 is also an RNA-binding protein that plays a role in the life cycle of many DNA and RNA viruses. During viral infection and cellular stress, activation of the innate immune response causes a redistribution of the cytoplasmic HNRNPA1 process. HNRNPA1 is a trans-acting factor that binds to the 5′ untranslated RNA single strand of human rhinoviruses and influences virus translation [34].

The RNA-binding proteins RBM5, RBM28, RBM42, and RBM6, which are all members of the same protein family, as well play a key role in the network. Despite the lack of a direct link between RBM28 and the core network, these proteins appear to play a role in a variety of cellular mechanisms, including cell cycle arrest and apoptosis induction via pre-mRNA splicing of multiple target genes, including TP53. They appear to be critical for cancer cell transformation and progression prevention in a variety of cancers, including lung cancer [35].

In 75% of primary lung cancers, as well as prostate and breast cancer, a possible reduction in RBM5 availability, mostly localized in the cytoplasm, has already been described [36].

Reduced cytoplasmic availability of the Strubbelig-receptor family protein group (SRSF1, SRSF7, SRSF9) can determine autophagy suppression regulated by SRSF1 and apoptosis of colon and lung cancer cells induced by SRSF7 and SRSF9 reduction [37–39].

Other factors, such as LIN28A (Lin-28 homolog A), FXR2 (FMR1 autosomal homolog 2), CELF6 (CUGBP Elav-like family member 6), and RBFOX1 (RNA-binding Fox-1 homolog 1), are also reported in the core network (Fig. 2).

FXR2 is involved in RNA metabolism, neuronal plasticity, and muscle development, and LIN28A suppression has been shown to inhibit osteogenic differentiation and decrease the expression of several osteogenic genes [40].

CELF6 is associated with the pathogenesis of myotonic dystrophy by regulating muscle-specific alternative splicing (DM). Furthermore, CELF6 depletion promotes cell cycle progression, proliferation, and colony formation by modulating p21 gene expression [41]. Finally, RBFOX1 belongs to the Rbfox protein family, which are master regulators of gene networks involved in neurogenesis as well as mature neuronal functions. RBFOX1 has been linked to the regulation of gene networks that aid cell survival in stressful conditions [42, 43].

The central network is not directly correlated with PABPC5 (polyadenylate-binding protein 5), YBX2 (Y-box-binding protein 2), SAMD4A (sterile alpha motif domain containing 4A).

PABPC5 is involved in the regulation of mRNA metabolic processes in the cytoplasm, whereas YBX2 is a major constituent of messenger ribonucleoprotein particles and is involved in the regulation of the stability of germ cell mRNAs (mRNPs) [44, 45].

Finally, SAMD4A is a conserved RBP found in a variety of species, and it regulates gene translation and stability. SAMD4A has been linked to angiogenesis and tumor progression in breast cancer, and its low expression in human breast tumor tissues/cells has been linked to a poor prognosis and survival [46].

### miRNA signature enrichment analysis

The in silico analysis was extended using the TomTom tool to analyze the presence of RNA fragment sequences generated by the degradation of RBP-binding motifs, taking into account a possible delay in physiological degradation of these motifs, such as a protective influence of RBPs-binding on motifs. With RBP target motif fragment sequences in common, we detected 39 candidate host miRNAs. (The complete list of miRNA is shown in Table 1.). It is well recognized that a single miRNA can regulate the expression of multiple target mRNAs (up to 100 at once) and that each mRNA can be regulated by multiple miRNAs [47]. Only miRNAs with more than two possible mRNA targets, cytoplasmic localization, and experimental evidence of regulatory effect in protein expression were selected from the miRNA list using GeneCodis4 and Mienturnet.

**Fig. 3:**
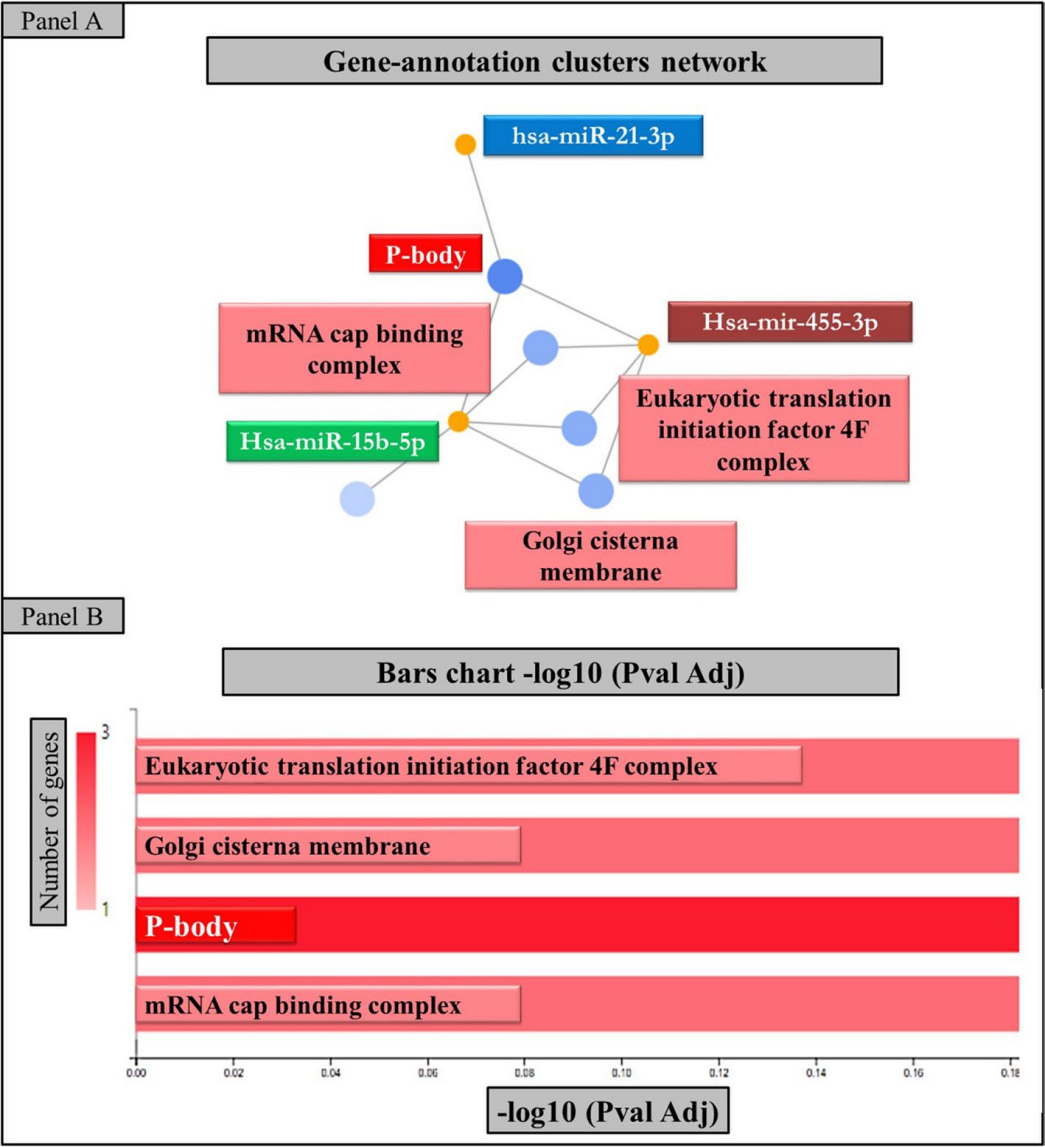
GeneCodis4 provided the network plots and bar chart results. Co-annotation and enrichment network plot (**A**). Only three miRNA interact with each other with a primary cytoplasmic localization as part of cytoplasmic ribonucleoprotein granules processing bodies, according to GeneCodis4 analysis of all 39 miRNA interactions (P-bodies). Each bar’s length corresponds to the −log10 (Adj. Pval) and the number of significant annotations found to agree with the color intensity (**B**)

**Fig. 4:**
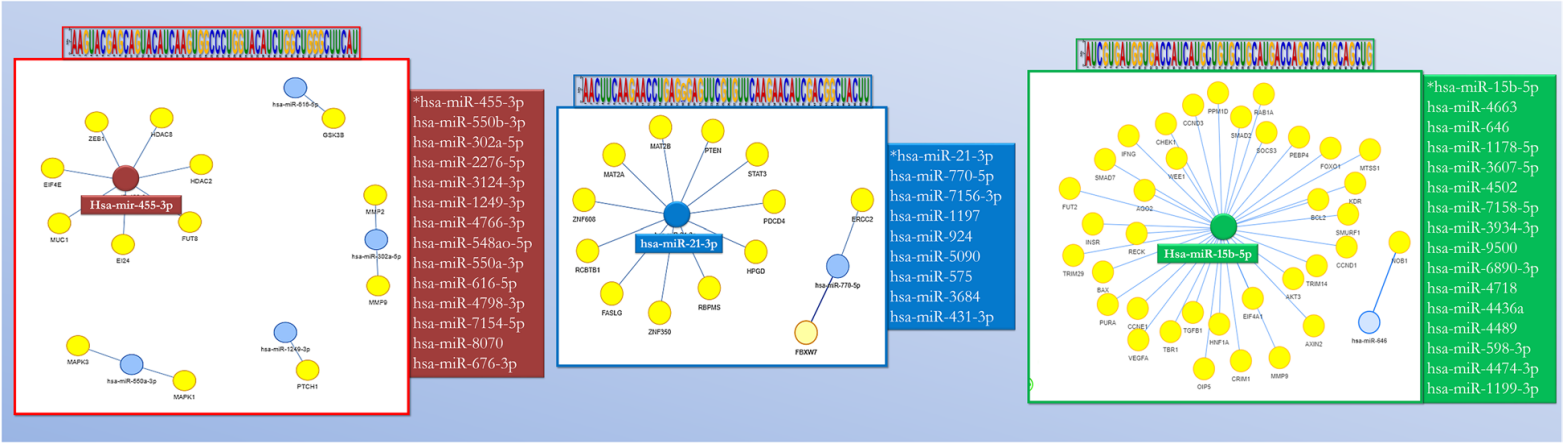
The output of MIENTURNET. The miRNAs that are similar to each motif are separated into boxes. The three miRNAs identified by GeneCodis4 analysis are highlighted in the networks

Only three miRNA (has-miR-455-3p, has-miR-31-3p, and has-miR-15b-5b) interact with each other with a cytoplasmic localization, in particular as part of cytoplasmic ribonucleoprotein granules in processing bodies (P-bodies) primarily composed of translationally repressed mRNAs (Figs. 3 and 4), indicating a regulatory effect in protein expression.

We discovered that the same three miRNA generated by GeneCodis4 are characterized by the greatest number of possible interactors, and thus can modify the expression of different mRNA, using the software Mienturnet on the same subset of 39 miRNA.

Finally, we looked into the possibility of has-miR-455-3p, has-miR-31-3p, and has-miR-15b-5b becoming more available in the cytoplasm.

In some cancers, Hsa-mir-455-3p plays an important role. Reduced levels of Hsa-mir-455-3p in pancreatic cancer cells reduce cell apoptosis while increasing cell migration, invasion, and EMT (epithelial-mesenchymal transition). Surprisingly, an increase in small RNA that mimics the effect of has-mir-455-3p may have the opposite and protective effect [48].

Furthermore, increased levels of hsa-miR-21-3p in hepatocellular carcinoma were found to be significantly elevated, indicating poor overall survival [49].

Finally, hsa-miR-15b-5p has been shown to either promote or inhibit tumor progression in a variety of tumor types. In breast cancer, hsa-miR-15b-5p expression was upregulated compared to normal breast tissue, and it was associated with poor overall survival in patients. In breast cancer-promoting breast cancer cell proliferation, migration, and invasion, hsa-miR-15b-5p availability significantly decreased HPSE2 expression at both the mRNA and protein levels [50].

## Discussion

In recent years, new evidence has been reported about the possible role of exogenous RNA in causing variations in cytoplasmic protein distribution, resulting in a wide range of structural, functional, and biochemical changes within host cells, furthering our understanding of the various “strategies” that RNA viruses can adapt to evade and suppress the innate immune system [51]. Even though the effects are discussed on a cellular level, the in silico study shows how viral RNA inside the cell can induce the subtraction of transcriptional factors (TFs) and potentially contribute to an explanation of clinical manifestation/symptomatology [15].

Over 250 new viral miRNAs were discovered at the same time, allowing researchers to investigate the function and biogenesis of virus-encoded miRNAs. Several studies have found that miRNAs encoded by RNA viruses have specific biological functions in gene regulation and the alteration of cellular pathways [52]. Based on this new evidence, we used a bioinformatics approach to find possible conserved RNA-binding domains motifs, which are recognized in a specific way by RBPs inside all RNA sequences coding for the SARS-CoV-2 S protein in representative variants and in three vaccines.

Furthermore, in silico analysis has been extended to assess the presence of specific sequences similar to human miRNAs in the same RBPs-binding motifs.

In the first hypothesis, we found conserved motifs targeted by host RBPs within the mRNA coding S protein and vaccine sequence, which could lead to abnormal protein recruitment. In this way, the impact of various cellular networks has been assessed.

In the second hypothesis, researchers looked into the possibility of RNA sequences similar to human mature miRNA being found within the same targeted RBPs motifs. Following the physiological degradation of exogenous RNA, the persistence of these RNA fragments (small non-coding RNAs) may be able to modulate various pathways.

The discovery of close interconnections between 17 of the 22 RBPs identified has led to the suggestion of an intricate network of RBPs (Fig. 2). RBPs’ primary function, as expected, is to regulate biological networks involved in the exogenous viral RNA stabilization process (MATR3, hnRNP family, FXR2, SAMD4A, YBX1). The involvement of YBX1 proteins in networks is of particular interest because these proteins are involved in a variety of oncogenic processes. The possible reduction of YBX1 cytoplasmic availability inhibits proliferation of myeloid differentiation-inducing apoptosis and decreases the expression of cyclin D1 and cyclin E1 associated with pancreatic ductal adenocarcinoma cell growth.

Reduced availability of the SRSF1 protein, which regulates autophagy activation, as well as SRSF7 and SRSF9, which induce apoptosis in colon and lung cancer cells by controlling apoptosis [27, 28].

Through pre-mRNA splicing of multiple target genes, including the tumor suppressor protein p53, RBM5, RBM28, RBM42, and RBM6, play a role in the induction of cell cycle arrest and apoptosis. These proteins have also been linked to the prevention of tumor transformation and progression in a variety of cancers, including lung cancer. RBM5 levels (mostly found in the cytoplasm) have been linked to the progression of 75% of primary lung cancers, as well as prostate and some breast cancers. Low expression of SAMD4A in human breast tumor tissues and cells was also linked to a poor prognosis.

The function of the HNRNPA1 protein is also intriguing because it has been described as an IRES trans-acting factor that binds the 5′ untranslated region of ssRNA human rhinovirus-2 and regulates its translation. The HNRNPA1 protein has been shown to have a high affinity for the SARS-CoV nucleocapsid protein (N) [45]. Blocking the availability of HNRNPA1 protein as a therapeutic approach for reducing viral translation has the potential to be effective. The evidence from our in silico investigation on specific small non-coding RNA sequences inside RBPs target motifs seems to support the involvement of YBX1, hnRNPs, and RBFOX1 in miRNA and pri-miRNA processing, as well as the regulation of gene networks that support cell survival during stress.

Other intricate connections capable of regulating different networks inside the host cell are highlighted by the in-depth in silico analysis. RBPs target motifs were analyzed, and 39 miRNAs with similar sequences to RBPs target motif were observed. We found that increased levels of miR-455-3p were associated with protective effects in pancreatic cancer cells by reducing cell migration and invasion, that hsa-miR-21-3p was significantly elevated in hepatocellular carcinoma and associated with poor overall survival, and that hsa-miR-15b-5p was associated with poor overall survival in breast cancer patients.

## Conclusion

The use of our pipeline allowed for the identification of highly conserved motifs capable of binding RBPs, reducing their cytoplasmic concentration, and inducing cellular system modifications in all known SARS-Cov-2 spike protein RNA sequences and then vaccine sequences.

The use of inexpensive and fast in silico analysis allowed for the provision of useful information that helped to focus the work in the wet lab. This approach is critical, especially in emergency situations like a pandemic, because it allows for rapid re-evaluation of new evidence (reverse genomics).

The results of this analysis provide fascinating insights into the possible variations in infected cellular functions, opening up new opportunities for understanding concurrent symptomatology and possible side effects during infections and vaccinations at the molecular level.

## Data Availability

The data that support the findings of this study are openly available.
